# Synthesis and Physiological Remodeling of CD34 Cells in the Skin following the Reversal of Fibrosis through Intensive Treatment for Lower Limb Lymphedema: A Case Report

**DOI:** 10.3390/dermatopathology10010016

**Published:** 2023-03-09

**Authors:** Jose Maria Pereira de Godoy, Ana Carolina Pereira de Godoy, Maria de Fatima Guerreiro Godoy, Dalisio de Santi Neto

**Affiliations:** 1 Department of the Medicine School, São José do Rio Preto (FAMERP), CNPq (National Council for Research and Development), Sao Jose do Rio Preto 15090-000, Brazil; 2 Research Group of the Clínica Godoy, Intensive Surgery Pediatric Cardiac, Hospital da Criança e Maternidade—HCM, Medicine School of Sao Jose do Rio Preto (FAMERP), Sao Jose do Rio Preto 15090-000, Brazil; acp.godoy@gmail.com; 3 Research Group in the Clínica Godoy, Medicine School of São José do Rio Preto (FAMERP), Sao Jose do Rio Preto 15090-000, Brazil; mfggodoy@gmail.com; 4 Hospital de Base, Medicine School of São José do Rio Preto (FAMERP), Sao Jose do Rio Preto 15090-000, Brazil; dalisio@gmail.com

**Keywords:** skin, CD34, telocytes, treatment, fibrosis

## Abstract

A novel type of cell underwent identification between 2005 and 2008 and was denominated the “telocyte” in 2010. In 2012, transmission electron microscopy revealed the presence of telocytes in the dermis. The aim of the present study was to report important changes in immunostained CD34 cells following the treatment of lower limb lymphedema using a specific lymphatic therapy technique. A clinical trial involving the evaluation of changes in immunostained CD34 cells in the epidermis and dermis (10 randomly selected histological fields) of a patient before and after intensive treatment for clinical stage II lymphedema was conducted using the Godoy Method, which was adapted to the treatment of skin fibrosis. The evaluation involved the use of the Weibel multi-point morphometric method. Comparisons were performed using the *t*-test with a 95% significance level. An important increase in CD34 cells was found with redistribution occurring following treatment. The treatment of primary lymphedema of the lower limbs resulted in the clinical reversal of fibrosis and an increase in the number of immunomarked CD34 cells.

## 1. Introduction

The first descriptions of telocytes (TCs) emerged between 2005 and 2008 among groups of Romanian and Italian researchers, who initially proposed the name “interstitial Cajal-like cells”. In 2010, researchers determined that this was a novel type of cell, so it was then denominated a “telocyte”, a cell type found in numerous organs [1,2,3]. Telocytes are cells with a small body and a variable number of prolongations known as telopodes, the form of which depends on the quantity present [1]. Although not specific, the two main markers of these cells are CD34 and PDGFRα. Transmission electron microscopy is one of the main techniques used for the anatomic identification of TCs, which are organized in networks, suggesting that this is a condition for the performance of their functions [4,5].

Initial research on telocytes proposed that these cells play roles in structural support and mechanical sensing, cell-to-cell signaling by interacting with many other cell types, and regulation of the immune response, although these functions remain to be proven experimentally. Recently, telocytes (TCs) were shown to function as crucial components of the stem cell niche [6,7,8]. In relation to telocytes in the vascular system, one study showed that telopodes surround the abluminal side of the lymphatic endothelial marker, suggesting a possible role of TCs in the regulation of lymphatic capillary functionality, which should be investigated further [9].

TCs coexist in the interstice of nearly all organs. In the occurrence of pathological loss, fibroblasts increase in number and produce a large quantity of extracellular matrices (ECMs), disorganizing the integrity of tissues [10]. One study suggested that TCs synthesize ECMs under exposure to hormonal stimuli [4]. These cells interact functionally with neighboring cells, producing exosomes (vesicles of different sizes) and transporting intercellular biological signals [4,10]. Mechanical cell-to-cell connections through contiguous plasma membranes form intercellular communication sites [11]. Several functions of these cells have been identified, such as their role in the synthesis of different molecules, as progenitors of mesenchymal cells, immunomodulation, regulation of the parenchyma (growth, maturation, and differentiation of adjacent cells), the induction of angiogenesis, scaffold support for other cells, and phagocytic properties [6]. In addition, studies on the vascular system have shown increasing evidence to suggest that TCs are morphologically or numerically destructed in fibrosis-related diseases, so TCs are associated with chronic inflammation and fibrosis. It is important to further study the potential roles of TCs in the onset and progression of systemic fibrosis diseases to improve the therapeutic outcomes of related diseases [12].

In 2012, a study involving transmission electron microscopy revealed the presence of TCs projecting specific telopodes in the dermis. The cutaneous TCs were found to be closely related to, or in contact with, fibroblasts, mastocytes, adipocytes, and bands of collagen and elastic fibers [11]. TCs are distributed in particular spatial relations with other structures (vessels, nerves, stem cells, and immunologically reactive cells) [13,14]. The normal density and distribution of TCs are affected by pathological conditions of the skin, such as psoriasis and systemic sclerosis [14,15]. Moreover, the normalization of their number is correlated with the remission of disease. These data lend support to the hypothesis that TCs participate in repair processes in skin tissue [16].

The aim of the present study was to report important changes in immunostained CD34 cells during the treatment of lower limb lymphedema using a specific lymphatic therapy technique.

## 2. Method

### 2.1. Patient and Setting

This study involved a patient from the Vascular Surgery Service of the public hospital affiliated with the Medicine School of São Jose do Rio Preto-FAMERP, SP, Brazil, Clínica Godoy, Institute of Biosciences and Exact Sciences, UNESP, Campus São Jose do Rio Preto, Biology Department—IBILCE Multiuser Center for Microscopy and Microanalyses. It was conducted in 2020.

### 2.2. Design

We clinically evaluated changes in the immunostained CD34 cells in the epidermis and dermis (10 randomly selected histological fields) of a patient before and after intensive treatment for clinical stage II lower limb lymphedema using the Godoy Method, which was adapted for the treatment of skin fibrosis. The evaluation involved the use of the Weibel multipoint morphometric method. Comparisons were performed using the *t*-test with a 95% significance level.

### 2.3. Inclusion Criteria

The inclusion criteria were the presence of clinical stage II lower limb lymphedema with intense fibrosis characterized by the absence of Godet’s sign.

### 2.4. Exclusion Criteria

The exclusion criteria were the presence of an active infection or edema due to other causes that had been diagnosed clinically.

### 2.5. Ethical Considerations

This study received approval from the institutional review board of the São Jose do Rio Preto School of Medicine (#4.398.518). The patient signed the consent form.

### 2.6. Statistical Treatment

The data were submitted to the normality test. Mean and standard deviation values were compared using the *t*-test with a 95% significance level.

### 2.7. Development

A 67-year-old male patient had received a clinical diagnosis of late primary lymphedema 12 years prior to participation in this study and reported an episode of erysipelas during this period. Other clinical causes of edema, such as kidney disease, heart disease, and hypoproteinemia, were ruled out. The physical examination revealed intense fibrosis characterized by the absence of Godet’s sign. Volumetry (a water displacement method) revealed a difference of 1200 g in comparison with the contralateral limb. Biopsies were performed prior to treatment and after the clinical normalization of the skin through intensive treatment using the Godoy Method^®^. For this, the patient was submitted to asepsis and antisepsis conditions. Local anesthesia was given with 2 mL of 2% xylocaine, and a longitudinal incision of approximately 1 cm in length by 0.5 cm in width in a wedge shape was made. The material was kept in a 10% formal solution and embedded in paraffin. The slides were immunostained for CD34 cells and examined under an optical microscope. Ten histological fields were randomly selected. The multipoint morphometric method proposed by Weibel was used. The data for these parameters are relative and expressed as percentages. Comparisons were made using the *t*-test with a 95% significance level.

### 2.8. Treatment

Intensive treatment was performed using the Godoy Method [17], which consists of cervical lymphatic therapy (approximately 30 gentle movements on the skin in the supraclavicular region for 15 to 20 min per day), [18] eight hours per day of mechanical lymphatic therapy using an electromechanical device that performed approximately 25 passive plantar flexion and extension movements per minute [19], two hours per day of manual lymphatic therapy [20], and a compression mechanism, which involved the use of a laced, hand-crafted grosgrain (non-elastic) stocking [21] alternated with medium-stretch elastic bandages that was maintained throughout the entire treatment process. Treatment lasted for two months and achieved clinical reversal of the fibrosis with an improvement in the elasticity of the skin. At this time, the follow-up biopsy was performed.

## 3. Results

**Figure 1:**
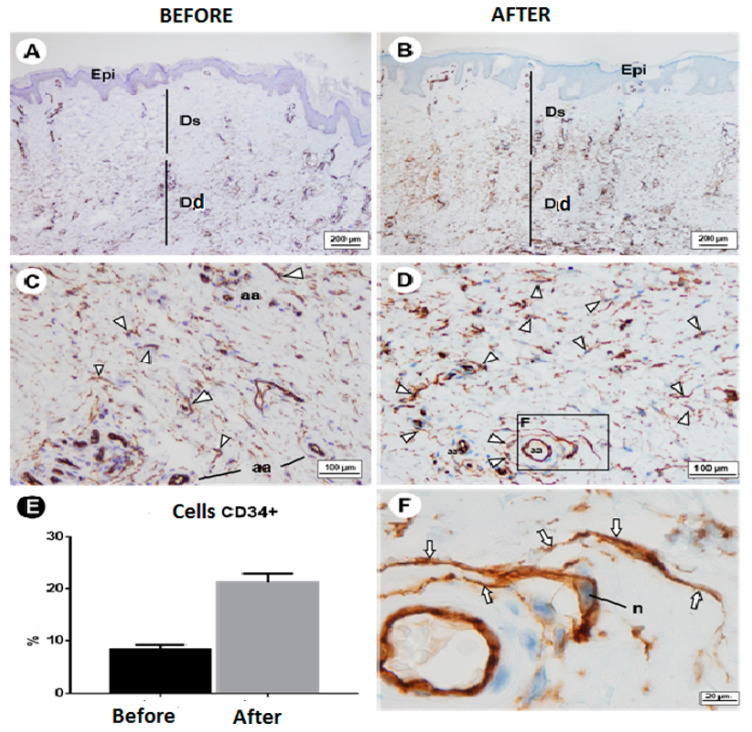
CD34+ cells in dermis. (**A**,**B**) Dermis before treatment, with immunostaining for CD34+ cells restricted to deep dermis. (**C**,**D**) Population of these cells increased in superficial dermis after treatment. Note the increase in immunostained cells mainly in the perivascular region and their dispersal in connective tissue. (**E**) Graph showing percentage increase in CD34+ cells. (**F**) Perivascular CD34+ cells. Note prolongation of this type of cell (arrows) around arterioles and that they are connected to each other (before; after; relative CD34+ cells; Dd). Abbreviations and symbols: aa: arterioles Epi: epidermis; Ds: superficial dermis; Dd: deep dermis; arrowhead: CD34+ cell; n: nucleus.

A significant increase in C34+ cells was found, going from a baseline of 8.30 ± 0.89% to 21.23 ± 1.64% after treatment, an increase of 236% (Table 1). Figure 1A,B show that the CD34+ cells in the dermis were restricted to the deep dermis prior to treatment. Figure 1C,D shows that the population of these cells in the superficial dermis increased following treatment. This increase occurred more in the perivascular regions, and the cells dispersed into the connective tissue. Prolongations of this type of cell were observed around arterioles and in connection to each other.

## 4. Discussions

The present study shows that the fibrotic process that occurs in the dermis of patients with lower limb lymphedema causes important changes in CD34 cells. The stimulation of the lymphatic system through intensive treatment led to the clinical reversal of fibrosis with the return of the elasticity of the skin and a significant 236% increase in the percentage of CD34+ cells in the dermis. These cells were initially restricted to the deep layer of the dermis. Following treatment, the cells were also found in the superficial layer, distributed in perivascular regions, and dispersed in the connective tissue. This is the first study to assess immunostained CD34 cells in a case of dermal fibrosis resulting from primary lower limb lymphedema.

The literature contains few studies on telocytes in the skin. However, changes in the density and normal distribution of these cells, as well as changes in the normal ultrastructure, have been observed in two pathological conditions: systemic sclerosis and psoriasis [14,15]. TCs are organized to form three-dimensional networks among collagen bundles and elastic fibers and around microvessels, nerves, and structures associated with the skin (pilous follicles, sebaceous glands, and sweat glands) [15,16].

The pattern of ultrastructural abnormalities in TCs in cases of systemic sclerosis (cytoplasmic vacuolization, swelling of the mitochondria, lipofuscin bodies) differs from that seen in psoriasis, which is characterized by important dystrophic changes (fragmentation of telopodes, cytoplasmic disintegration, apoptotic nuclei, nuclear extrusions) [22]. These changes were found in this case of primary lymphedema, and the clinical reversal of fibrosis through specific treatment led to an increase in the number of telocytes as well as normal redistribution in the dermis. Skin affected by scleroderma undergoes progressive rupture of the dermal network of telocytes until the cells are nearly completely lost in the advanced/fibrotic stage of skin disease [1].

The improvement in the number of TCs is correlated with the remission of disease, thereby suggesting the participation of these cells in the skin repair process [15]. In studies with animal models, wounds treated with exogenous telocytes were shown to have an accelerated healing process as well as a reduction in the inflammatory response induced by lipopolysaccharides (LPSs), with the restriction of apoptosis and migration of the main cell types present in the skin [23].

In another study, immunofluorescence and confocal laser scanning microscopy revealed two subpopulations of dermal TCs; one expressed c-kit/CD117 and the other was positive for CD34 [24]. Both subpopulations were also positive for vimentin. In the present investigation, an evaluation of CD34 cells was performed, suggesting the need for further evaluation of these markers in future studies.

Different physiopathological mechanisms have been found regarding telocytes in psoriasis, scleroderma, and lymphedema, which may affect the characteristics of the lesions and the clinical stage of the disease. The clinical reversal of psoriasis is associated with the normalization of telocytes, as occurred in the present study in a case of lymphedema. In psoriasis, the inflammatory process leading to fibrosis is the main physiopathological cause of the event. Therefore, treatment involves the reversal of inflammation. In lymphedema, the physiopathology is the accumulation of macromolecules, which is reversed with treatment. Thus, the different physiopathological processes may contribute to the evolution of fibrosis and changes in telocytes.

Telocytes are injured under fibrotic conditions in different organs found in diseases such as Crohn’s disease, ulcerative colitis, hepatic fibrosis, and primary Sjögren syndrome [20]. Stimulation of the lymphatic system was employed in the present study and may be useful for the treatment of these diseases [25]. Thus, our results may pave the way for an important line of research on fibrotic and inflammatory processes.

The treatment used in this study showed an important alteration in the proteins of the extracellular matrix involving type I and type III collagen and elastic fibers [26,27]. The development of the fibrotic process in patients with lymphedema involves the accumulation of macromolecules in the interstitial space, and this evolves with changes in extracellular matrix proteins. Telocytes alterations were identified in the study. Thus, these cells and the relationships they have with extracellular matrix proteins in the skin should be investigated. The fibrotic process that occurs in lymphedema is an important structural change that can be reversed with specific treatment. The importance of each protein fiber in the evolution of this process and in the remission of the disease, as well as their relationships with telocytes and their changes, can bring about specific telocytes responses during the evolution of lymphedema.

This study suggests that transplanting telocytes into damaged tissue can promote tissue regeneration. Therefore, increasing telocytes in tissue through transplantation or promoting their survival and growth using new drugs represents a therapeutic strategy that could be used in the future [28]. The present study involved the stimulation of the lymphatic system with specific manual techniques. The possibility of combining new ways of stimulating TCs could contribute to the treatment of lymphedema, therefore opening an important line of investigation.

In different skin diseases, different patterns of disruption of the structure and ultrastructure of TCs are observed. The cooperation of telocytes with other interstitial elements and their immunological profile and changes during disease remission suggest that these cells play a role in tissue regeneration/repair processes. [29] Further research should be carried out to improve our knowledge on telocytes and to identify the mechanisms involved in the self-repair of the skin. The present study shows that self-repair of the skin can be encouraged through the use of specific techniques to stimulate the lymphatic system, suggesting the importance of the lymphatic system in this transformation.

A study on a mouse model of bleomycin-induced scleroderma was used to evaluate the evolution of TCs in the fibrotic process of the skin. CD34 immunofluorescence revealed severe impairment in the dermal network of telocytes/CD34+ stromal cells. Another study on animals showed that, during the development of fibrosis, telocytes undergo important transformations, whereas the present study demonstrated the regeneration of fibrotic processes and the marked improvement in the telocyte concentration [30].

A study reported that, in the vascular system, telocytes form a three-dimensional network and establish direct contact with blood vessels, budding endothelial cells, and active macrophages, exerting their effect through paracrine signaling. VEGF expression by CTs, endothelial cells, and macrophages is necessary for endothelial cell proliferation and migration and vascular growth. Macrophages can facilitate phagocytosis and the elimination of degraded components of the ECM [31]. Relationships of telocytes with blood vessels and macrophages and the control of cell degradation products were observed in this study.

Another study reported that the distribution of CD34+SCs/TCs around blood and lymphatic vessels in the skin varies depending on the size, location, and type of vessel. All skin vessels, except those in the papillary dermis, are surrounded by CD34+SCs/TCs, which are an important component of the adventitia of larger vessels and form a boundary layer in most of the smaller ones [32].

The presence of the cells associated with these vessels shows their importance in the circulatory system, but specific research is required to identify their exact functions. The present study shows that the regeneration of these cells interferes with the stimulation of the lymphatic system in skin fibrosis. The importance of lymphatic vessels in the evolution of all diseases that evolve with a fibrotic aspect has also been shown. In the vascular area, it has been reported that the concentration of TCs significantly increases in tissues of patients with chronic wounds compared to healthy controls. These cells reduce the delay in wound healing, and the inflammatory responses caused by liposaccharides can be mediated by inflammatory inhibition, thus restricting apoptosis and promoting the migration of the main types of skin component cells [23] Telocytes have officially existed in the literature for a few years, but today, their important role in the pathogenesis of multiple diseases is being identified. TCs have been suggested as new cellular targets for forthcoming therapies, and the development of specific methods in this area represents an important objective for science.

## 5. Conclusions

This study presented a case report of a patient undergoing treatment for primary lymphedema of the lower limbs. The treatment resulted in the clinical reversal of fibrosis and an increase in the number of immunomarked CD34 cells.

## Figures and Tables

**Table 1:** Morphometry of epidermis and dermal papillae and presence of C34+ cells before and after treatment (SEM).

	Before	After
**Morphometry**		
**CD34+ cells—Dermis (%)**	8.30 ± 0.89 ^b^	21.23 ± 1.64 ^a^

^a, b^ Different letters denote significant differences between evaluation times (*p* < 0.05).

## Data Availability

The data used to support the findings of this study are included within the article.
